# An applied scholarship framework for population health impact: adapting the Boyer model for public health faculty

**DOI:** 10.3389/fpubh.2026.1860100

**Published:** 2026-08-12

**Authors:** Vaughn Mamlin Upshaw, Karar Zunaid Ahsan, Daisy Naa Sowah, Rohit Ramaswamy, Weiming Tang

**Affiliations:** 1 Gillings School of Global Public Health, University of North Carolina at Chapel Hill, Chapel Hill, NC, United States; 2 The University of North Carolina at Chapel Hill School of Medicine, Chapel Hill, NC, United States

**Keywords:** academic promotion, implementation science, population health, public health faculty, scholarship, translation

## Abstract

Research-intensive (R1) schools of public health increasingly expect faculty to generate evidence that supports real-world implementation, workforce development, and policy-relevant decision-making for public benefit. Yet academic reward structures continue to privilege traditional discovery-focused outputs while underrecognizing the scholarly work required to translate evidence into practice. This misalignment weakens the connection between academic labor and public health impact. Achieving impact in practice requires trust, proximity to those closest to the problem, and contextual understanding. Translating evidence from controlled studies into diverse real-world settings demands both technical and adaptive skills and produces outputs not typically recognized in academic reward systems. Building on Boyer's influential model of discovery, integration, application, and teaching, we propose an updated Applied Scholarship Framework for R1 Research Universities with Schools of Public Health. The framework organizes faculty work across four domains—Inquiry, Translation, Implementation, and Learning and Improvement—and specifies evidentiary standards associated with each. Reframing applied work as rigorous scholarship rather than ancillary service better aligns faculty evaluation, institutional strategy, and public health accountability. Such a model can help schools of public health strengthen practice partnerships, support diverse faculty roles, and accelerate the movement of evidence into action.

## 1 Introduction

Schools of public health are increasingly being pushed to show how their work is having an impact on population health outcomes. For instance, the University of North Carolina at Chapel Hill's Gillings School of Global Public Health is ranked the top public school of public health in the United States, yet North Carolina continues to rank poorly on several major health indicators, including infant mortality, premature death, and the share of uninsured women (1). This leads to the question of “what difference are we making?” In response, a growing number of public health faculty and students are engaged in solving urgent problems, supporting implementation of evidence-based and new approaches in varied and complex systems, training the next workforce to respond to emerging threats, and partnering with communities and agencies to improve population health (2, 3). Despite this, academic reward systems governing many research-intensive universities (R1 in the Carnegie Classification of Institutions of Higher Education) remain oriented toward a narrower model of scholarly value—one that tends to privilege peer-reviewed publications and traditional grant-funded research over the applied, translational, implementation, and improvement work through which public health impact is often achieved.

This mismatch has consequences for public health. In response to growing demands for real-world impact, translation and implementation science have rightly expanded as promising fields within academia. Yet impact is ultimately realized in practice-based settings—through partnership work that adapts interventions to local constraints, builds implementation capacity, and generates learning from real-world use. Too often, such practice-based implementation is treated as the domain of health departments, delivery systems, and NGOs, while academic incentives channel much of the work into narrower research paradigms that culminate in publications more than sustained change on the ground. This dynamic is described as the research–practice gap paradox: implementation science produces a growing body of knowledge and tools, but a central question remains whether its primary audience becomes other researchers and whether the knowledge reaches beyond academia to practitioners (4). The result is a structural disconnect between what public health schools say they value, namely population health impact, and what their evaluation systems most consistently reward.

That disconnect matters at a time when public trust in scientific and public health institutions, including schools and programs of public health, is strained. Pew Research Center has documented skepticism toward scientific experts and growing partisan divides in trust in science (5). Gallup has reported declines in confidence in major US institutions, including science (6). Research!America polling has similarly shown that although many Americans continue to value science, fewer express confidence that scientists and public health officials act consistently in the public's best interest (7). In this environment, academic public health must do more than training public health professionals and produce publishable discoveries and new grants, it must also demonstrate trustworthiness, relevance, usefulness, accountability, and responsiveness to real-world conditions affecting communities (8).

Schools of public health require a more explicit and contemporary framework for recognizing applied scholarly work. Building on Boyer's seminal model in Scholarship Reconsidered: Priorities of the Professoriate (1990), we propose an Applied Scholarship Framework (ASF) for R1 public health faculty that more accurately reflects how knowledge is generated, translated, implemented, and improved in practice. This is not merely a faculty affairs concern; it is a public health systems issue, as academic incentives shape which forms of work are pursued, sustained, and legitimized. Although this mismatch is relevant to any academic institution engaged in practice-facing public health work, it is especially pronounced in R1 universities, where faculty are expected both to compete successfully for external research funding and to produce generalizable knowledge while also demonstrating meaningful population health impact, often within constrained institutional budgets that must also support faculty, staff, and core school infrastructure.

## 2 Boyer's model and its limits in contemporary public health

In his 1990 publication, Scholarship Reconsidered, Boyer advanced a highly influential framework that broadened the definition of scholarship beyond research and new discoveries. He articulated four contemporary forms of scholarship—discovery (generating new knowledge), integration (connecting ideas across disciplines and placing findings in a broader context), application (using knowledge to address real problems in practice), and teaching (systematic, reflective work to improve learning; see Table 1). This model has been widely adopted as a common vocabulary for faculty roles and, in many academic institutions, has informed how promotion and tenure guidelines describe and evaluate scholarly contributions beyond publications and grants. Boyer's central argument was that universities should “break out of the tired old teaching vs. research debate” and recognize “the full range of faculty talent,” creating conceptual space for a broader set of faculty contributions (9, p. xii). In 2023, Boyer continued his call for a more engaged academic model, emphasizing that both civic and academic vitality are strengthened when “scholars and practitioners speak and listen carefully to each other” (9).

**Table 1:** Boyer's scholarship priorities for professors (16).

Boyer's priorities of the professoriate	Functions
Scholarship of discovery	Creation of new knowledge through disciplined research
Scholarship of integration	Interpretation and synthesis of knowledge across disciplines to provide broader meaning and context
Scholarship of application	Responsible use of knowledge to address important real-world problems in ways that are rigorous and can also generate new understanding
Scholarship of teaching	Scholarly teaching that educates, stimulates learning, and extends knowledge

Since the initial publication, Boyer's expanded his thinking about the scholarship of integration across the academy stating there is “an urgent need to place discoveries in a larger context and create more interdisciplinary conversations” among the academic disciplines (10, p. 20). Moving engaged scholarship beyond the academy itself demands a new set of processes: translation of evidence for decision-making, implementation support in real systems, and embedded bi-directional learning through evaluation and adaptation. As a result, these forms of work often remain conceptually blurred and administratively undervalued.

This problem is reinforced by the way faculty evaluation systems operate in practice. Even where institutions endorse broad definitions of scholarship, review processes often default to conventional proxies of merit. Uzoka et al. (10) described broad acceptance of Boyer's framework alongside limited formal mechanisms for integrating its multiple dimensions into actual assessment systems. Helton and Pathman (11) similarly noted the need for clearer criteria to evaluate diverse scholarly products. Milner et al. (12) argued that scholarship definitions must evolve to reflect contemporary forms of academic contribution and their effects on practice and community.

In public health, the stakes of this misalignment are especially high. Faculty work often occurs in partnership with health departments, schools, community organizations, health systems, and policymakers. The value of that work depends not only on generating evidence but also on making it usable, implementable, and improvable in context. This reality suggests that Boyer's framework, while foundational, needs an applied update to capture how evidence becomes impact in modern public health settings. If academic systems fail to recognize those efforts as scholarship, they discourage precisely the forms of work most needed to improve population health.

It is our intention to offer a framework for applied scholarship in public health led by instructors who Donald Schön of MIT describes as “reflective practitioners” (10, p. 45). This framework explicitly describes how engaged public health scholarship uses practice-driven knowledge to shape research and translate its lessons from research so they can be implemented in practice. This creates a continual learning loop by promoting new discoveries and applications in the academy, fueled by community experience, while simultaneously funneling research findings and new approaches back into practice settings where they generate public benefits.

## 3 An applied scholarship framework for public health scholarship

Public health practice in the current era places demands on faculty roles that Boyer's original categories do not fully specify. Public health faculty are increasingly expected to generate evidence that their work is having an impact on real-world challenges, communicate their work to multiple audiences, support uptake of new methods in organizational and community settings, monitor equity and performance, and adapt interventions over time. These activities are not peripheral to public health; they are central to whether evidence produces real benefit.

To respond to these demands, we adapt Boyer's model in ways that preserve its core insight while making the categories more specific to contemporary public health work in research-intensive settings (see Table 2). We retain Discovery in the form of “Inquiry” as the traditional domain of rigorous curiosity that generates generalizable knowledge and methods (e.g., manuscripts, method products, validated measures). We then differentiate Boyer's integration and application to better reflect how public health faculty move evidence toward use. In the ASF, translation captures synthesis and communication of evidence for decision-making and adoption (e.g., briefs, guidance, toolkits with evidence statements, tailored dissemination), while Implementation captures the practical design and delivery of partner-facing tools, workflows, and capacity-building supports that enable use in real systems. Finally, we broaden Boyer's teaching domain into Learning & Improvement to emphasize that practice-based scholarship depends on embedded evaluation, quality assurance, equity monitoring, and iterative adaptation (“what changed and why”) and that the learning generated through these activities feeds back into how public health professionals are taught and trained. These refinements reflect a broader shift toward more inclusive scholarship frameworks in R1 contexts, while also acknowledging that translating recognition into consistent appointment, promotion, and tenure (APT) practice remains uneven, particularly for practice- and clinical-track faculty (13) and often depends on institutional support structures that can help make diverse products legible and reviewable (14, 15).

**Table 2:** Comparing priorities from Boyer and applied scholarship framework for public health.

Boyer's scholarship model		Applied scholarship framework	
Priorities	Defined	Priorities	Defined
Scholarship of discovery	Through research, extend the frontiers of human knowledge	Inquiry	Through rigorous methods uncover generalizable findings
Scholarship of integration	Through interdisciplinary conversations, place discoveries in larger context	Translation	Through synthesis of research generate practical evidence for decision-making and adoption
Scholarship of application	Through use, make scholarship relevant to human ends	Implementation	Through adaptation and co-design support delivery in context
Scholarship of teaching	Through publication and teaching, communicate with students and peers	Learning and Improvement	Through iterative feedback, adapt how public health professionals are taught and trained

**Figure 1:**
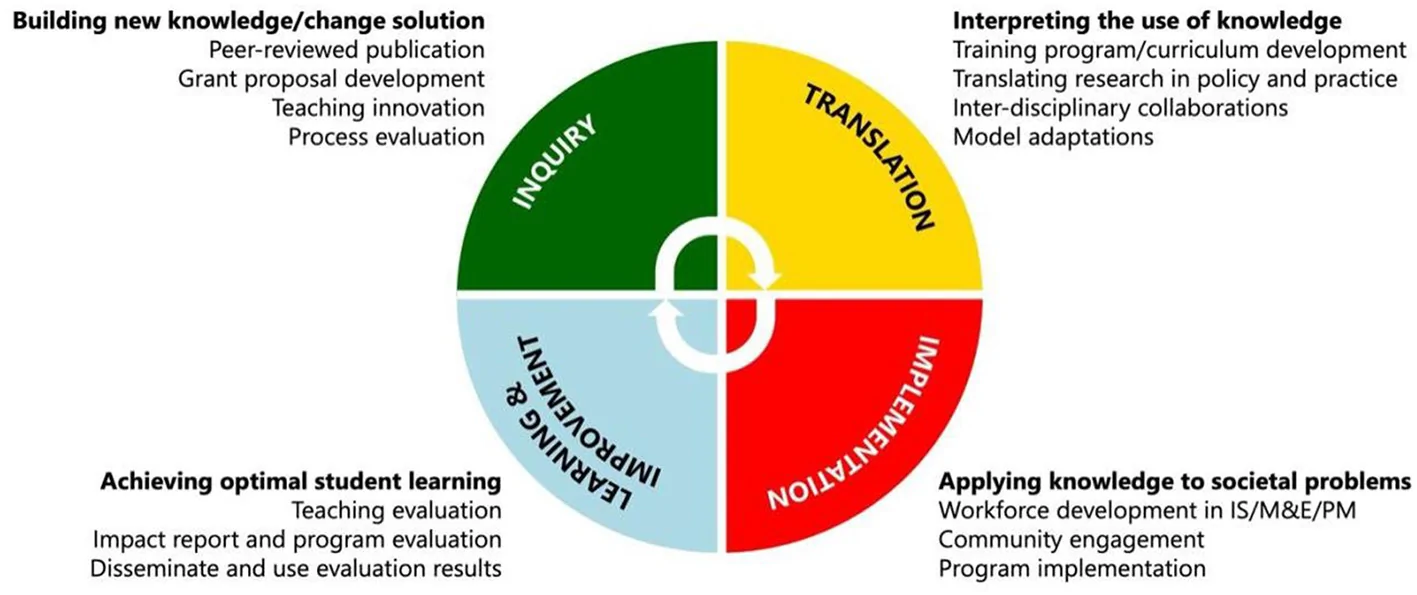
Applied scholarship framework. IS, implementation science; M&E, monitoring and evaluation; PM, program management.

Accordingly, the ASF for public health faculty in research-intensive universities organizes faculty work into four interrelated domains: inquiry, translation, implementation, and learning and improvement. Together, these domains reflect a continuous cycle that links knowledge generation to practical use and iterative refinement (see Figure 1).

### 3.1 Inquiry

Inquiry refers to the generation of new generalizable knowledge driven by the priorities identified by engaging with communities of practice in their contexts. Applied scholars use rigorous methods to support inquiry into the problems affecting communities, organizations, and networks required to address complex public health issues. Grounded scholarship includes traditional research activities such as developing interventions, methods, models, frameworks, theories, metrics, and analytic approaches (9, 16, 17), as well as public health activities such as community health assessments (CHAs) and community-driven assets, infrastructure, capacity and urgent priorities. Products include community profiles, geographic maps, health assessments, peer-reviewed articles, validated measures, data sets, codes, and funded research proposals. Standards for evaluating inquiry remain familiar: scientific rigor, quality, transparency, reproducibility, peer review, and contribution to the field.

### 3.2 Translation

Translation focuses on synthesizing, adapting, and communicating evidence so that observations from research and practice can be turned into decisions and interventions that improve health (18, 19). Public health faculty routinely convert research findings into forms that practitioners, policymakers, educators, and organizations can use. Products generated through translation include extracting and/or synthesizing findings from the literature using approaches such as systematic reviews or comparative analysis, to produce delivery protocols, and/or applied methods adapted for use by public health practitioners. Comparative policy analysis, customized best-practices, and tailored recommendations for stronger monitoring are examples of scholarly deliverables that facilitate decision-making, and priority-setting. These deliverables can be consistently assessed using standards for evaluating scientific rigor and scholarship within the academy.

### 3.3 Implementation

Implementation in scholarship supports the use of evidence in real-world systems. Faculty in this domain may collaborate with public agencies, health systems, or community organizations to adapt interventions, build organizational capacity, and support sustained uptake. Products may include implementation of toolkits, playbooks, protocols, workflows, training packages, and implementation studies. Understanding fidelity through process evaluation should emphasize feasibility, usability, reach, partner-verified uptake, and implementation outcomes such as adoption, adaptation, and sustainability (20, 21).

### 3.4 Learning and improvement

Learning and improvement captures embedded evaluation, quality assurance, equity monitoring, and iterative adaptation in real-world settings, as well as the translation of those lessons into teaching and training for the public health workforce (22–24). Public health interventions rarely function exactly as designed when introduced into complex systems. This domain recognizes the scholarly value of documenting what changed, why it changed, and what was learned through adaptation, including how those insights inform curricula, training materials, and capacity-building approaches. Products in this domain may include policy briefs, evidence syntheses, improvement reports, learning briefs, guidance documents, training materials and teaching resources, standardized instruments and measures, and decision-support tools. Scholarship in this domain should be judged not only by accuracy and clarity but also by contextual fit and documented use in decisions, plans, guidance, or program design.

Evaluation standards should include methodological appropriateness, transparency about limitations, and evidence that learning informed meaningful improvement in practice and workforce development.

## 4 From product counts to evidence bundles

A central contribution of the ASF is that it shifts evaluation away from narrow product counts and toward evidence bundles. An evidence bundle is a curated set of materials that demonstrates the quality of a contribution and documents its use across one or more ASF domains. It typically combines the scholarly output itself with documentation of uptake or implementation, partner or stakeholder validation, and evidence of learning, adaptation, or outcomes.

For example, an evidence bundle for an Implementation-domain contribution might combine a partner-facing toolkit or workflow (the scholarly product) with a brief process-evaluation memo documenting fidelity and reach, a short letter or email from the partnering agency confirming adoption and use, and a one-page summary of adaptations made and lessons learned. Taken together, these materials enable reviewers to evaluate quality and impact with the same rigor applied to a peer-reviewed manuscript, without requiring the product itself to take the form of a publication.

This approach emphasizes the continuum of diagnosis, design and delivery of interventions, policies or programs (25). Public health faculty generate many outputs beyond manuscripts, and those outputs can be rigorously assessed when reviewers look for evidence that fulfills one or more of the following:

- meeting specific project goals,
- enhancing partner capability to deal with similar problems into the future,
- generating new ideas and insights that strengthen outreach capabilities and contribute to the knowledge base of the field,
- impacting teaching and research for the individual and their colleagues,
- benefiting student learning and engaged practice, and contributing to the mission of the institution and the unit [adapted from Lynton (25), p. 35]

Evidence may be evaluated based on agreed standards for product quality, including community feedback, peer and/or expert review, documented use, reach across settings, partner validation, and contribution to improved decisions, implementation performance, or outcomes. This approach aligns with Helton and Pathman's (11) observation that scholarship can be assessed across diverse product types using criteria such as expertise, contribution, originality, peer review, quality, permanence, and impact. This orientation resonates with broader research-assessment reform efforts such as the Declaration on Research Assessment (26), which similarly calls for evaluating scholarly contributions on their merits rather than through narrow, metric-based proxies.

Elevating the science and rigor needed to translate, adapt and implement research to improve public health outcomes is particularly important when the triple aim for the academy is typically described as “teaching, research, and service.” Applied research is often difficult to distinguish from service under academic guidelines. Service to one's profession as a volunteer may support applied scholarship, such as when individuals serve as reviewers, program planners, or board members for professional organizations, but it becomes applied scholarship when those roles generate reviewable scholarly outputs that are used by the organization or other stakeholders, along with evidence of the outputs' quality and use and how they contribute to improved public benefits. In schools and programs for public health, applied scholarship engagement is often reflected in the institutional mission but absent from expectations and incentives for faculty performance. Articulating the value of applied scholarship, which delivers products for practitioners and community partners rather than for academic journals, as a valuable academic contribution that differs from service makes visible the intellectual labor embedded in community engagement, partnership development, implementation support, and iterative improvement that is necessary to improve public health systems and outcomes.

## 5 Why this matters for public health impact

The case for an applied scholarship framework is not only conceptual; it is also practical and political. Public and private institutions of higher education, and schools of public health in particular, have an obligation to advance research that promotes public benefit. Decades of research have shown which factors contribute to premature death and disease, yet rates of noncommunicable diseases continue to increase around the world. When academic institutions explicitly reward deliverables that drive real-world change, they shape the kinds of work faculty pursue, how departments allocate labor, and what institutions signal as valuable. When promotion systems privilege inquiry over implementation, peer-reviewed publications over applied products, or formal theory over real-world evidence, they constrain the translational and implementation work needed to improve public health outcomes and contribute to the continued underuse of effective interventions described in the literature. Public health impact depends on more than generating evidence. It depends on whether evidence is translated for decision-makers, adapted to context, implemented with both fidelity and flexibility, and improved over time. Moving research into practice is an applied scholarly challenge, not merely an administrative task.

An ASF also has implications for workforce development. Schools of public health are responsible for preparing graduates to work across sectors and systems, yet R1 faculty incentive structures often undervalue the applied scholarship, practice engagement, teaching, mentoring, and partnership work necessary to build that workforce. Recognizing applied scholarship can help align faculty labor with the educational and civic missions of public health institutions.

In ASF, service is operationalized through “evidence bundles.” ASF distinguishes clearly between (1) service roles that are necessary to sustain academic programs and public health institutions but do not inherently produce scholarly outputs, and (2) service-related work that generates reviewable outputs used by stakeholders and therefore qualifies as applied scholarship. To reduce ambiguity and avoid inflating routine service into scholarship, ASF uses a pragmatic documentation rule: service without reviewable outputs is recorded as a yes/no contribution, often with a simple hierarchy reflecting the immediacy of institutional need, typically departmental, then school, then external; service that produces outputs with documented quality and use can be included as scholarship within the appropriate ASF domain(s) and supported by an evidence bundle.

In addition, such a framework may strengthen public accountability. In a period of mistrust, academic public health must show not only that it produces knowledge, but also that it contributes to decision-making, systems improvement, and equitable outcomes. Rewarding scholarship that is visible, useful, and responsive to communities may help institutions better demonstrate public value. At the same time, reward system reform cannot be considered in isolation from the broader funding environment: extramural support substantially shapes which projects faculty pursue and sustain, and adjustments to promotion and tenure criteria must remain attentive to institutional and organizational viability, not only to internal alignment between mission and metrics.

## 6 Equity, partnership, and institutional accountability

An updated scholarship framework should also place equity more centrally within academic evaluation. Historically, universities have often engaged communities primarily as sites of data collection rather than as partners in knowledge production. The ASF emphasizes health equity through co-production, reciprocity, and accountability in research and practice partnerships. It also promotes equity in faculty evaluation by making the applied scholarly products and evidentiary standards associated with community-engaged participatory research, applied policy work, implementation science, and related applied fields more explicit and reviewable across the academy. The rationale for using an evidence bundle, rather than relying solely on traditional and narrowly defined research outputs, is to better capture how equity can be reflected in faculty work: through the selection of problems that elevate practical challenges in the field, through forms of productivity that value applied outputs, and through a more balanced conception of scholarship that recognizes both traditional research and applied scholarship.

This matters because public health inequities are shaped by structural conditions that cannot be addressed through discovery alone. Faculty members who work with communities and agencies to adapt interventions, monitor distributional effects, and improve implementation in underserved settings are producing knowledge that is essential to advancing health equity. Yet that work often remains less visible in conventional academic review systems.

Broadening scholarship criteria will not automatically produce equity. Institutions must also invest in who has access to partnership opportunities, mentoring, and dissemination channels by providing the support and infrastructure needed to develop and sustain high-quality applied work. Without such investments, expanded criteria may reproduce existing inequities across research and scholarship approaches rather than reduce them. Still, a more explicit ASF provides a basis for evaluating whether academic labor aligns with core public health values, including equity, reciprocity, and measurable benefits for communities. It also strengthens public accountability by encouraging evidence that partnership-based work was used, improved practice or performance, and reduced inequities in reach, implementation, or outcomes.

## 7 Implications for R1 schools of public health

For research-intensive universities, schools of public health, and related disciplines, adopting an ASF has several implications. Faculty in the Department of Public Health Leadership and Practice (PHLP) at UNC Gillings developed and adopted the ASF as a foundation for faculty appointment, promotion, and tenure (APT) beginning in 2026. Guidelines for faculty productivity and evaluation under the ASF apply across all faculty tracks and ranks within PHLP to ensure consistent and equitable recognition of faculty contributions. Although not every department has updated its APT guidelines to prioritize applied scholarship deliverables, school-wide APT policies and university review processes were recently revised to allow faculty to advance using these updated criteria.

Our experience with this revised APT guideline has led to several actions moving forward. The actions below reflect anticipated benefits that are currently being evaluated as part of the piloted adoption of the refined ASF at UNC Gillings, rather than empirically confirmed outcomes.

First, promotion and tenure criteria can may explicitly recognize products across all four domains, including translational outputs, implementation tools, and learning artifacts. Evaluation should rely on bundles of evidence rather than on single metrics. Adopting consistent definitions, guidelines, evaluation criteria, and expected deliverables across units, while also providing explicit guidance to external reviewers, can facilitate adoption, learning, and adaptation across broader institutional contexts.

Second, schools are may be better positioned to support pluralistic faculty roles. Public health academic institutions need inquiry-focused researchers, but they also need faculty who specialize in implementation, community engagement, workforce development, and applied improvement. A broader framework provides a basis for recruiting practice-oriented scholars, legitimizing their work, and improving retention of faculty whose contributions are essential to ensuring that research findings generate public benefits, strengthen capacity, foster shared learning, and bring real-world experience into the classroom and the training of future public health professionals.

Third, schools can may better value and commit to long-term community-academic partnerships with health departments, health systems, and community organizations as core infrastructure rather than peripheral service. These partnerships have often been framed narrowly as sites for research, but they must also support practice-facing work that enables translation, implementation, and improvement in real-world settings. Supporting faculty, students, and professional liaisons in community settings, governmental agencies, nonprofit organizations, and health care institutions fosters trust, which in turn increases engagement from communities of practice in shared inquiry, translation, implementation, and learning and improvement. These partnerships enable communities to shape research, policy, programs, workforce development, and applied innovation. Partnerships with trusted communities should be supported as long-term institutional investments, much like investments in laboratories and technologies.

Finally, schools adopting the ASF are may be better positioned to align their internal strategies for applied scholarship with their institutional missions and, more importantly, with core public health values and principles. If institutions value impact, equity, and community engagement in their missions, then their evaluation systems should reward the work required to achieve those goals. Revisiting the alignment among institutional mission statements, professional values, and faculty incentives is essential to ensuring that these policies and processes equitably recognize and reward applied scholarship across disciplines and units.

Although we developed the ASF for public health faculty, its underlying logic is not specific to our field. Any applied discipline in which faculty partner with organizations and communities outside the academia, such as social work, nursing, environmental science, education, and urban planning, among others, confronts a similar mismatch between practice-facing work and conventional scholarship metrics. The four ASF domains, and the evidence-bundle approach used to document them, could be adapted to these fields by substituting discipline-specific products and evidentiary standards for the public health examples described here.

## 8 Conclusion

Public health challenges increasingly require rapid movement of evidence into action across complex systems. Yet academic reward structures have not kept pace with these demands. Schools of public health continue to rely heavily on evaluation systems that privilege inquiry while underrecognizing the scholarly work of translation, implementation, and iterative improvement.

Updating Boyer's framework through an ASF offers a practical way to better align faculty evaluation with public health mission. By recognizing Translation, Implementation, and Learning and Improvement as legitimate scholarly domains alongside Inquiry-driven advances, research-intensive schools of public health can better support the work required to improve population health.

More broadly, this shift would represent not just a refinement of academic evaluation but a reorientation of academic public health toward greater accountability for real-world impact. At a moment when public trust is fragile and health challenges are increasingly complex, that reorientation is both institutionally necessary and publicly urgent.

## Data Availability

The original contributions presented in the study are included in the article/supplementary material, further inquiries can be directed to the corresponding author.
